# Supplementary material for: Choline Metabolism to the Proatherogenic Metabolite Trimethylamine Occurs Primarily in the Distal Colon Microbiome In Vitro
Source: Metabolites. 2025 Aug 16;15(8):552. doi: 10.3390/metabo15080552 (PMC12388646; doi:10.3390/metabo15080552)
Supplement: Supplementary file 1 [file metabolites-15-00552-s001.zip › metabolites-3750334-supplementary.pdf]

Supplementary Information

for

Choline metabolism to the proatherogenic metabolite trimethylamine occurs primarily in the distal regions of the MiGut 3-compartment *in vitro* colon microbiome model

Supplementary Figure 1. *MiGut figure*

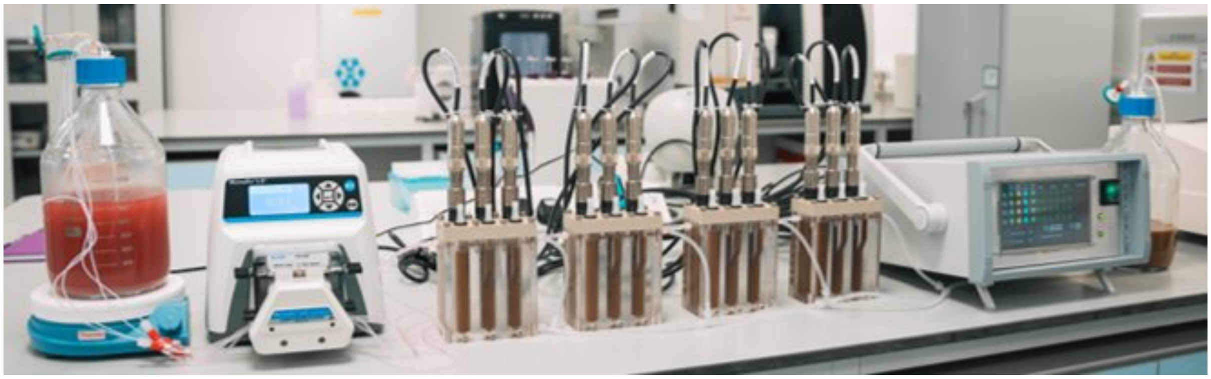

Supplementary Figure 1. MiGut setup. A single human MiGut *in vitro* platform consisting of 4 independent triple-staged models. All environmental parameters are measured and controlled by the controller unit on the right and fed with the media outlined in Supplementary Table 1 on the left.

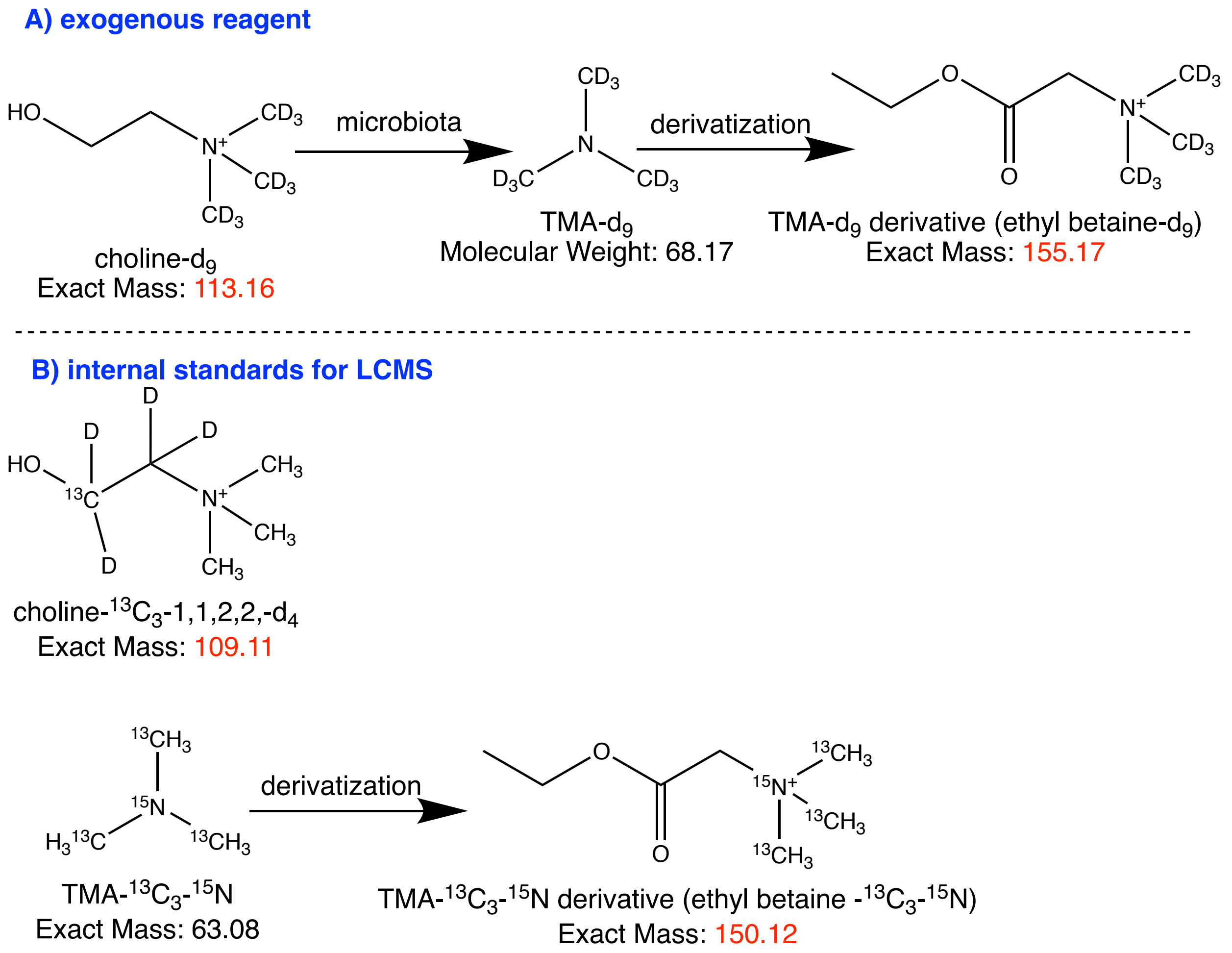

***Supplementary Figure 2**. A) Experimental reaction and analyte derivatization. B) Internal standards and derivatization.*

Supplementary Figure 3. *MiGut microbiome composition.*

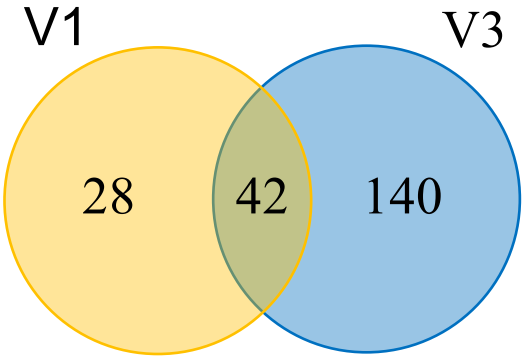

Supplementary Figure 3. Shared and unique bacterial genera in MiGut. Metagenomic analysis of microbial genera in vessel 1 (proximal colon; yellow circle) and vessel 3 (distal colon; blue circle). Numbers represent the shared and unique microbial taxa, at the genera level, within the MiGut model.

Supplementary Table 1. *Media composition for MiGut*

Media Component	Weight/volume
Magnesium sulphate	0.01 g/L
Calcium chloride	0.01 g/L
Sodium chloride	0.1 g/L
Di-potassium monohydrogen phosphate	0.04 g/L
Potassium di-hydrogen phosphate	0.04 g/L
Sodium hydrogen carbonate	2.0 g/L
Haemin	0.005 g/L
Cysteine HCL	0.5 g/L
Bile Salts	0.5 g/L
Arabinogalactan	1.0 g/L
Tween 80	0.2 %
Pectin	2.0 g/L
Starch	3.0 g/L
Vitamin K1	10 μL/L
Peptone Water	2.0 g/L
Yeast extract	2.0 g/L
Chenodeoxycholic acid	0.25 g/L
Lithocholic acid	0.017 g/L
Mucin	4 g/L
Resazurin (post-autoclaving)	0.005 g/L
*Glucose (post-autoclaving)	0.4 g /L

*Glucose included in the media for the first week only after faecal slurry added to the model.

Supplementary Table 2. *Microbial taxa identified in vessels 1 and 3*

Vessel 1 species			
Algiphilus sp. NNCM1	Clostridium sp. AM32-2	Lactobacillus acidophilus	Ralstonia sp.
Anaerofilum sp. BX8	Clostridium sp. TM06-18	Lactobacillus crispatus	Roseburia hominis
Bacteriophage sp.	Collinsella aerofaciens	Lactobacillus delbrueckii	Salmonella enterica
Bacteroides fragilis	Coprococcus catus	Lactobacillus gallinarum	Schleiferilactobacillus harbinensis
Bariatricus massiliensis	Enterobacter hormaechei	Lactobacillus gasseri	Schleiferilactobacillus perolens
Bifidobacteriaceae bacterium	Enterocloster bolteae	Lactobacillus helveticus	Schleiferilactobacillus shenzhenensis
Bifidobacteriaceae bacterium	Enterocloster citroniae	Lactobacillus hominis	Shigella boydii
Bifidobacterium adolescentis	Enterocloster clostridioformis	Lactobacillus iners	Shigella dysenteriae
Bifidobacterium animalis	Enterococcus faecalis	Lactobacillus jensenii	Shigella flexneri
Bifidobacterium bifidum	Enterococcus faecium	Lactobacillus johnsonii	Shigella sonnei
Bifidobacterium breve	Escherichia albertii	Lactobacillus paragasseri	Siphoviridae sp.
Bifidobacterium catenulatum	Escherichia coli	Lactobacillus taiwanensis	Staphylococcus aureus
Bifidobacterium dentium	Escherichia fergusonii	Lentilactobacillus parabuchneri	Streptococcus agalactiae
Bifidobacterium longum	Faecalibacterium prausnitzii	Ligilactobacillus ruminis	Streptococcus pyogenes
Bifidobacterium longum	Faecalibacterium sp.	Limosilactobacillus antri	Streptococcus thermophilus
Bifidobacterium pseudocatenulatum	Gardnerella vaginalis	Limosilactobacillus fermentum	Veillonella atypica
Bifidobacterium pseudocatenulatum	Haemophilus influenzae	Limosilactobacillus oris	Veillonella dispar
Brugia timori	Hungatella hathewayi	Limosilactobacillus reuteri	Veillonella parvula
Campylobacter jejuni	Intestinimonas butyriciproducens	Lupinus albus	Veillonella sp.
Candida albicans	Klebsiella pneumoniae	Megasphaera micronuciformis	Veillonella sp. ACP1
Candida dubliniensis	Lacticaseibacillus paracasei	Myoviridae sp.	Veillonella sp. DORA_A_3_16_22
Caudovirales sp.	Lacticaseibacillus rhamnosus	Oscillibacter sp.	Veillonella sp. ICM51a
Chlamydia trachomatis	Lactiplantibacillus paraplantarum	Pediococcus acidilactici	Veillonella tobetsuensis
Citrobacter koseri	Lactiplantibacillus pentosus	Podoviridae sp.	uncultured Clostridium sp.
Clostridium sp. AM22-11AC	Lactiplantibacillus plantarum	Pseudoalteromonas sp. S558	uncultured bacterium
**Vessel 3 species**			
Megasphaera vaginalis	Chlamydia trachomatis	Firmicutes bacterium CAG:129_59_24	Oscillibacter sp. PC13

Akkermansia muciniphila	Christensenella minuta	Firmicutes bacterium CAG:176	Oscillibacter valericigenes
Alistipes communis	Christensenellaceae bacterium	Firmicutes bacterium CAG:176_63_11	Oscillospiraceae bacterium
Alistipes finegoldii	Citrobacter koseri	Firmicutes bacterium CAG:24	Parabacteroides distasonis
Alistipes indistinctus	Clostridia bacterium	Firmicutes bacterium CAG:83	Parabacteroides goldsteinii
Alistipes onderdonkii	Clostridiales bacterium	Firmicutes bacterium TM09-10	Parabacteroides merdae
Alistipes shahii	Clostridiales bacterium 1_7_47FAA	Flavobacteriales bacterium	Parabacteroides sp. 20_3
Alistipes sp. CAG:29	Clostridiales bacterium 42_27	Flavonifractor plautii	Parabacteroides sp. D13
Allobacillus salarius	Clostridiales bacterium 52_15	Fournierella massiliensis	Pediococcus acidilactici
Anaerobutyricum hallii	Clostridiales bacterium 59_14	Frisingicoccus caecimuris	Phascolarctobacterium succinatutens
Anaerofilum sp. BX8	Clostridioides difficile	Fusicatenibacter sp. CLA-AA-H277	Phocaeicola vulgatus
Anaerofustis stercorihominis	Clostridium hathewayi CAG:224	Gemmiger formicilis	Phocea massiliensis
Anaerotignum lactatifermentans	Clostridium sp. AF50-3	Holdemania filiformis	Podoviridae sp.
Anaerotignum propionicum	Clostridium sp. CAG:138	Holdemania massiliensis	Pseudoalteromonas sp. S558
Anaerotruncus colihominis	Clostridium sp. CAG:169	Hungatella effluvii	Pseudoflavonifractor capillosus
Anaerotruncus massiliensis	Clostridium sp. KLE 1755	Hungatella hathewayi	Pseudomonas aeruginosa
Anaerotruncus rubiinfantis	Clostridium sp. MCC353	Hungatella sp. L12	Ralstonia sp.
Anaerotruncus sp. 22A2-44	Clostridium sp. MD294	Hydrogenoanaerobacterium saccharovorans	Roseburia hominis
Anaerovorax odorimutans	Clostridium tertium	Intestinibacillus massiliensis	Roseburia intestinalis
Bacteriophage sp.	Clostridium transplantifaecale	Intestinimonas butyriciproducens	Roseburia sp. CLA-AA-H209
Bacteroides acidifaciens	Collinsella aerofaciens	Intestinimonas massiliensis	Ruminococcaceae bacterium AF10-16
Bacteroides caccae	Coprococcus catus	Klebsiella pneumoniae	Ruminococcaceae bacterium TF06-43
Bacteroides cellulosilyticus	Coprococcus comes	Lachnoclostridium pacaense	Ruminococcus bovis
Bacteroides cellulosilyticus CAG:158	Desulfovibrio piger	Lachnospiraceae bacterium	Ruminococcus bromii
Bacteroides faecis	Desulfovibrionaceae bacterium	Lachnospiraceae bacterium 3_1_57FAA_CT1	Ruminococcus gauvreauii
Bacteroides finegoldii	Dialister pneumosintes	Lachnospiraceae bacterium AM48-27BH	Ruminococcus sp. 1001270H_150608_F2
Bacteroides fragilis	Dorea formicigenerans	Lachnospiraceae bacterium NLAE-zl-G231	Ruminococcus sp. AF16-40
Bacteroides intestinalis	Dorea longicatena	Lachnospiraceae bacterium NSJ-29	Ruminococcus sp. AF42-10
Bacteroides oleiciplenus	Dysosmobacter sp. BX15	Lachnospiraceae bacterium TF09-5	Ruminococcus sp. AF43-11
Bacteroides ovatus	Dysosmobacter sp. Marseille-Q4140	Lachnotalea sp. AF33-28	Ruthenibacterium lactatiformans

Bacteroides salyersiae	Eggerthella lenta	Lacrimispora sp. 210928-DFI.3.58	Salmonella enterica
Bacteroides sp. 2_2_4	Eisenbergiella massiliensis	Lacticaseibacillus rhamnosus	Schleiferilactobacillus harbinensis
Bacteroides sp. 3_1_23	Eisenbergiella porci	Lactiplantibacillus plantarum	Schleiferilactobacillus perolens
Bacteroides sp. D2	Eisenbergiella sp. OF01-20	Lactobacillus acidophilus	Schleiferilactobacillus shenzhenensis
Bacteroides stercoris	Eisenbergiella tayi	Lactobacillus gasseri	Shigella boydii
Bacteroides thetaiotaomicron	Emergencia timonensis	Lactobacillus hominis	Shigella dysenteriae
Bacteroides uniformis	Enterobacter hormaechei	Lactobacillus johnsonii	Shigella flexneri
Bacteroides xylanisolvens	Enterocloster aldenensis	Lactobacillus paragasseri	Shigella sonnei
Bariatricus massiliensis	Enterocloster asparagiformis	Lactobacillus taiwanensis	Siphoviridae sp.
Bifidobacterium adolescentis	Enterocloster bolteae	Lawsonibacter sp. NSJ-51	Streptococcus pyogenes
Bifidobacterium animalis	Enterocloster citroniae	Lentilactobacillus parabuchneri	Stylonychia lemnae
Bifidobacterium bifidum	Enterocloster clostridioformis	Lentisphaerae bacterium ADurb.Bin242	Subdoligranulum sp. APC924/74
Bifidobacterium breve	Enterocloster lavalensis	Limosilactobacillus fermentum	Sutterella seckii
Bifidobacterium catenulatum	Enterococcus durans	Limosilactobacillus mucosae	Sutterella sp. 63_29
Bifidobacterium longum	Enterococcus faecalis	Limosilactobacillus oris	Sutterella sp. AM11-39
Bifidobacterium longum CAG:69	Enterococcus faecium	Limosilactobacillus reuteri	Sutterella sp. KLE1602
Bifidobacterium pseudocatenulatum	Escherichia albertii	Lizonia empirigonia	Sutterella wadsworthensis
Bifidobacterium pseudolongum	Escherichia coli	Longicatena caecimuris	Tyzzerella sp. An114
Bilophila sp. 4_1_30	Escherichia fergusonii	Mailhella massiliensis	Veillonella atypica
Bilophila wadsworthia	Eubacterium sp. CAG:581	Megasphaera micronuciformis	Veillonella tobetsuensis
Bittarella massiliensis	Evtepia gabavorous	Megasphaera vaginalis	Vescimonas coprocola
Blautia obeum	Faecalibacterium prausnitzii	Methylocella tundrae	Wickerhamomyces anomalus
Blautia producta	Faecalibacterium sp.	Mogibacterium kristiansenii	[Clostridium] hylemonae
Blautia wexlerae	Faecalibacterium sp. CLA-AA-H233	Mogibacterium sp. BX12	[Clostridium] innocuum
Butyricicoccus sp. AM27-36	Faecalibacterium sp. Marseille-P9590	Myoviridae sp.	[Clostridium] scindens
Campylobacter gracilis	Faecalicatena contorta	Ogataea polymorpha	[Clostridium] symbiosum
Campylobacter jejuni	Faecalicoccus pleomorphus	Oscillibacter sp. 57_20	[Eubacterium] rectale
Candida africana	Firmicutes bacterium AF16-15	Oscillibacter sp. CAG:155	[Ruminococcus] gnavus
Candida albicans	Firmicutes bacterium AF36-19BH	Oscillibacter sp. ER4	[Ruminococcus] torques
Candida dubliniensis	Firmicutes bacterium CAG:103	Oscillibacter sp. MCC667	

Catenibacillus scindens	Firmicutes bacterium CAG:114	Oscillibacter sp. MSJ-31	
Caudovirales sp.	Firmicutes bacterium CAG:124	Oscillibacter sp. NSJ-62	
